# Analysis of Reporting Trends of Serious Adverse Events Associated With Anti‐Obesity Drugs

**DOI:** 10.1002/prp2.70080

**Published:** 2025-02-24

**Authors:** Branislava B. Raičević, Andrej Belančić, Nikola Mirković, Slobodan M. Janković

**Affiliations:** ^1^ University of Kragujevac, Faculty of Medical Sciences Kragujevac Serbia; ^2^ Department of Basic and Clinical Pharmacology and Toxicology University of Rijeka, Faculty of Medicine Rijeka Croatia

**Keywords:** anti‐obesity drugs, semaglutide, serious adverse drug events, trends analysis

## Abstract

Concern over the side effects of anti‐obesity medications, particularly if severe, has grown as their use has increased. Thus, the objective was to use trends in the reporting of suspected adverse events associated with anti‐obesity medications that have been approved for sale in the European Union to attempt to uncover discrepancies in the safety of these medications. The study was designed as secondary research, based on data about the number of adverse drug reactions (both serious and non‐serious) reported to the EudraVigilance database. Trends of the annual reporting rates for the six anti‐obesity drugs were analyzed by the Joinpoint Trend Analysis Software that divides the trendline into an optimum number of segments connected by “joinpoints” and tests the significance of the trend within each segment. The trends of serious adverse drug events showed clear differences among the anti‐obesity drugs: while all drugs had significant increasing trends during a few initial years after their appearance on the market, only the annual number of reports for semaglutide continued to grow ever since (annual change + 67.1%, *p* = 0.000). On the contrary, a continuous increase in the reporting rate of non‐serious adverse drug events was observed only for liraglutide (annual change + 33.8%, *p* = 0.000) while for the other anti‐obesity drugs, including semaglutide, the trends after the initial period were either negative or did not increase significantly. In conclusion, among the anti‐obesity drugs currently approved, only semaglutide shows a continuously increasing trend in the annual reporting of serious adverse events, suggesting a need for further investigation of safety signals.

## Graphical abstract

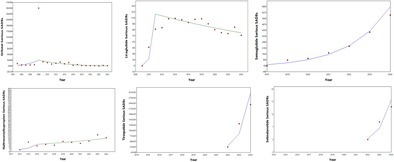

## 1 Introduction

Although numerous substances were used for the treatment of obesity in the past [1], currently, there are six active principles having marketing authorization in countries of the European Union as anti‐obesity medications: orlistat, semaglutide, liraglutide, naltrexone/bupropion, tirzepatide, and setmelanotide. The long‐acting glucagon‐like peptide‐1 receptor agonists (GLP‐1RAs) liraglutide and semaglutide are also potent anti‐diabetic agents. In patients with diabetes mellitus Type 2, liraglutide and semaglutide slow gastric emptying, stimulate glucose‐dependent insulin secretion, improve beta‐cell function, and decrease secretion of glucagon, leading to improved control of glycemia together with weight loss [2, 3]. Anti‐obesity drugs are increasingly prescribed (in the United States of America anti‐obesity drugs were prescribed to 1.1% of obese persons in 2010 and to 2.9% in 2019) [4], especially for women [5], and up to 8% of patients after bariatric surgery continue to use anti‐obesity drugs [6]. European countries also witness a continuous increase in anti‐obesity drug prescribing: from 1999 to 2006, the annual prevalence of anti‐obesity drug prescriptions in the United Kingdom increased 15‐fold [7], and from 2017 to 2022 in Norway, 2.8% of the adults were prescribed an anti‐obesity drug. In 2022, 1.04% of the adults in Norway used bupropion‐naltrexone, 0.91% used liraglutide, and 0.68% semaglutide [8]. About 10% of Italian endocrinologists prescribe liraglutide as the main treatment for their obese patients [9].

Parallel with increasing utilization of anti‐obesity drugs, there is growing concern about their adverse effects. A recent systematic review of clinical trials with anti‐obesity drugs found higher rates of drug discontinuation due to adverse effects with naltrexone‐bupropion, glucagon‐like peptide—1 receptor agonists, and orlistat than with other anti‐obesity drugs [10]. Analysis of the FDA Adverse Event Reporting System (FAERS) databases for the period 2013–2020 showed that as many as 18,675 reports of adverse events were associated with anti‐obesity drugs prescribed to 15,143 patients [11]. It is especially worrying that more than 90% of adverse events associated with anti‐obesity drugs and spontaneously reported to the European adverse events database were characterized as serious [12].

Many aspects of the adverse effects of anti‐obesity drugs remain unclear, especially when serious but rare adverse effects are in question. Recently, one of the anti‐obesity drugs (lorcaserin) was withdrawn from the market due to its association with increased occurrence of cancer, but it took more than 8 years of lorcaserin's widespread use to recognize the extent of the risk [13]. Analyzing trends of reporting adverse events of some drugs (e.g., of antidepressants) was helpful for the recognition of signals ultimately leading to an update of their safety profile [14]. When the increasing trend of spontaneous adverse drug reaction reports in Germany from 2000 to 2019 was compared with trends of the German population as a whole and the subpopulation of children, it was possible to identify changes in the administrative regulation of spontaneous reporting as a likely cause [15].

Our research's objective was to use trends in the reporting of suspected adverse events associated with anti‐obesity medications that have been approved for sale in the European Union to attempt to uncover discrepancies in the safety of these medications.

## 2 Materials and Methods

The study was designed as secondary research, based on data about the number of adverse drug reactions (both serious and non‐serious) reported to the EudraVigilance—European database of suspected adverse drug reports, established and maintained by the European Medicines Agency (EMA) from 2002 onward [16]. Since ethics committee approval was not necessary for this kind of research, it was not requested.

The suspected adverse drug reports were analyzed for six drugs that have the treatment of obesity as one of the approved indications in countries of the European Union: orlistat, semaglutide, liraglutide, naltrexone/bupropion, tirzepatide, and setmelanotide. The outcome variables in the study were the counts of serious adverse drug reactions (SADRs) and non‐serious adverse drug reactions (nSADRs) per calendar year (annual reporting rate), starting from the moment when reporting to EudraVigilance started. The independent variable was the reporting year. The counts of both SADRs and nSADRs are aggregate data, including all spontaneous reports in the database, regardless of the adverse drug reaction type; such an approach implies that the figures represent more overall attention of prescribers that these drugs captured than certain specific adverse effect mechanisms.

Trends of the annual reporting rates for the six drugs were analyzed by the Joinpoint Trend Analysis Software, version 5.2.0, issued and maintained by the National Cancer Institute of the USA [17, 18]. This is the regression software that divides the trendline into an optimum number of segments connected by “joinpoints”, and tests the significance of the trend within each of the segments. The significance of the trends is tested by a Monte Carlo Permutation method, and the annual percent change of the rates is calculated. The significance level was set at ≤ 0.05 probability of the null hypothesis.

## 3 Results

**FIGURE 1:**
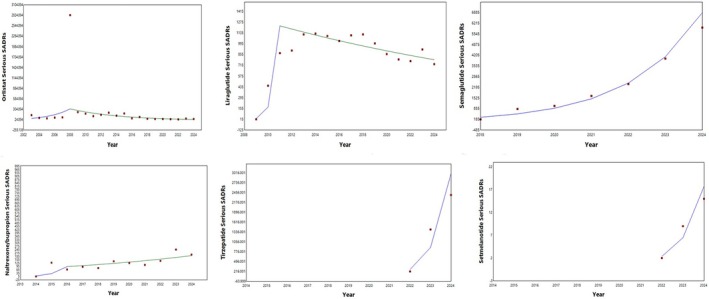
Trends in reporting serious adverse events associated with anti‐obesity medications.

**FIGURE 2:**
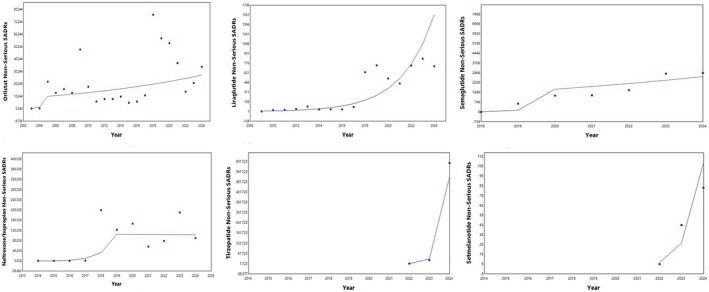
Trends of reporting non‐serious adverse events associated with anti‐obesity medications.

Trends of counts of suspected adverse drug reactions (SADR) per year were analyzed for six anti‐obesity drugs: orlistat, semaglutide, liraglutide, naltrexone/bupropion, tirzepatide, and setmelanotide, for the years 2002–2024, depending on the availability of the data in Eudravigilance—European database. The data were extracted from the database on September 16th, 2024. The trends of serious SADRs are shown in Figure 1, and those of non‐serious SADRs are presented in Figure 2. The annual percent change of the counts and the significance of the trends for each drug are shown in Tables 1 and 2 (serious SADRs and non‐serious SADRs, respectively).

**TABLE 1:** Trend segments, annual percent change, and significance of the trend segments for Serious Suspected Adverse Drug Reactions (SADRs).

Medicine	Trend segment	Annual percent change (%)	*p* value
Orlistat	1	41.3	0.080
	2	−14.1	**0.019**
Liraglutide	1	611.8	**0.000**
	2	−3.4	0.124
Semaglutide	1	67.1	**0.000**
Naltrexone/Bupropion	1	203.8	**0.007**
	2	9.4	0.689
Tirzepatide	1	232.6	**0.000**
Setmelanotide	1	173.9	**0.000**

**TABLE 2:** Trend segments, annual percent change, and significance of the trend segments for Non‐Serious Suspected Adverse Drug Reactions (SADRs).

Medicine	Trend segment	Annual Percent Change (%)	*p* value
Orlistat	1	512.4	**0.012**
	2	5.3	0.402
Liraglutide	1	281.1	0.227
	2	33.8	**0.000**
Semaglutide	1	1079.0	**0.000**
	2	11.6	0.917
Naltrexone/Bupropion	1	215.5	**0.003**
	2	−0.3	0.914
Tirzepatide	1	1621.9	**0.000**
Setmelanotide	1	307.4	**0.000**

Trends for serious SADRs of orlistat, liraglutide, and naltrexone/bupropion exhibited two segments each, the initial one showing a sharp increase for all drugs, and the second showing either a decrease or insignificant increase for orlistat, liraglutide, and naltrexone/bupropion. Trends for serious SADRs of semaglutide, tirzepatide, and setmelanotide had only one segment, sharply and significantly increasing, but data for only 3 years were available for the last two drugs (Figure 1 and Table 1). Trends for non‐serious SADRs of orlistat, liraglutide, semaglutide, and naltrexone/bupropion also exhibited two segments each, the initial one showing a sharp increase for all drugs, and the second showing either a decrease or insignificant increase for orlistat, semaglutide, and naltrexone/bupropion, while for liraglutide, the second trend segment was significantly increasing. Trends for non‐serious SADRs of tirzepatide and setmelanotide also had only one segment, sharply and significantly increasing, due to limited data availability (only 3 years) (Figure 2 and Table 2).

Available proprietary preparations of orlistat, semaglutide, liraglutide, naltrexone/bupropion, tirzepatide, and setmelanotide, their marketing authorization dates, and onset of SADRs reporting to Eudravigilance are shown in Table 3. Furthermore, the most frequently reported AEs associated with anti‐obesity drugs are presented in Table 4.

**TABLE 3:** Regulatory and SADRs reporting data for six antiobesity drugs.

Proprietary/non‐proprietary name	Therapeutic area	ATC code	Marketing authorization date	First reporting of SADRs	Total SADRs
Saxenda	Obesity; Overweight	A10BJ02	23/03/2015	07/06/2015	5843
Victoza	Diabetes Mellitus, Type 2	A10BJ02	30/06/2009	09/08/2009	1 2450
Lilraglutide (INN)		A10BJ02		29/01/2009	18922
Mysimba	Obesity; Overweight	A08AA	26/03/2015	21/03/2017	880
Naltrexone/bupropion (INN)		A08AA		12/01/2014	2236
Alli (previously Orlistat GSK)	Obesity	A08AB01	22/07/2007	14/08/2007	1100
Xenical	Obesity	A08AB01	29/07/1998	24/01/2003	3603
Orlistat (INN)		A08AB01		24/01/2003	5380
Ozempic	Diabetes Mellitus	A10BJ06	08/02/2018	22/02/2018	18649
Rybelsus	Diabetes Mellitus, Type 2	A10BJ06	03/04/2020	01/09/2020	3435
Wegovy	Obesity; Overweight	A10BJ06	06/01/2022	01/12/2022	1579
Semaglutide (INN)		A10BJ06		01/03/2018	2 6727
Imcivree	Obesity	A08AA	16/07/2021	15/06/2022	159
Setmelanotide (INN)		A08AA		15/06/2022	159
Mounjaro	Diabetes Mellitus, Type 2; Obesity; Overweight	A10BX16	15/09/2022	22/07/2022	4327
Mounjaro Kwikpen		A10BX16	15/09/2022	23/04/2024	60
Tirzepatide (INN)		A10BX16		22/07/2022	4631

**TABLE 4:** Most frequently reported adverse events (AEs) associated with anti‐obesity drugs.

Drug	The most frequently reported adverse drug reactions
Orlistat	Fatty/oily stool, abdominal pain, abdominal distension, increased defecation, respiratory infection, headache, hypoglycemia
Semaglutide	Nausea, vomiting, diarrhea, abdominal pain, constipation, decreased appetite, hypoglicemia, fatigue
Liraglutide	Nausea, vomiting, diarrhea, abdominal pain, constipation, insomnia, hypoglicemia, rash
Naltrexone/Bupropion	Anxiety, insomnia, headache, tremor, dysgeusia, somnolence, tinnitus, palpitations, nausea, constipation, dry mouth, hyperhidrosis, fatigue
Tirzepatide	Nausea, vomiting, abdominal pain, constipation, diarrhea, flatulence, hypersensitivity reactions, hypoglicemia, fatigue, hair loss
Setmelanotide	Injection site reactions, hyperpigmentation, spontaneous erections, nausea, vomiting, headache, polydipsia, back pain, pruritus, depression

## 4 Discussion

The trends of serious adverse drug events showed clear differences among the anti‐obesity drugs: while all drugs had a significant increasing trend during a few initial years after their appearance on the market, only the annual number of reports for semaglutide continued to grow ever since. The reporting rates for the other five anti‐obesity drugs were either decreasing or without significant increases. On the contrary, a continuous increase in the reporting rate of non‐serious adverse drug events was observed only for liraglutide, while for the other anti‐obesity drugs, including semaglutide, the trend after the initial period was either negative or did not increase significantly.

One of the possible causes of the continuous increase in the reporting rate of serious adverse events associated with semaglutide use could be a constant increase in utilization, which was observed in some countries. In the United States of America, semaglutide was the third top‐selling drug in 2023 and became the first top‐selling drug in 2024 [19, 20]; semaglutide prescriptions increased in numbers by 442% between January 2021 and December 2023 [21]. However, in Europe, where this study was conducted, liraglutide and naltrexone/bupropion were prescribed more frequently than semaglutide in 2022: 1.04% of adults in Norway were using naltrexone/bupropion, 0.91% liraglutide, and only 0.68% semaglutide [8]. Besides, if the continuously increasing trend of serious adverse events associated with semaglutide was a consequence of increased prescribing, one could expect that non‐serious adverse events would be increasingly reported, too; but this was not the case in our study.

When an increasing trend of utilization of certain drugs is associated with a decreasing trend of reporting serious adverse events, this points to a good safety profile of such drugs and precludes the necessity for further observational pharmacovigilance studies, except for spontaneous reporting. An increasing trend of utilization of drugs for attention deficit hyperactivity disorder was observed in England while reporting of their serious and fatal ADRs dropped by 1.79% per year for the whole group except for guanfacine, which showed a 40% annual increase [22]. A similar situation was observed for metformin and new oral anticoagulants, whose utilization increased, but the adverse events reporting rate decreased [23, 24]. However, if the opposite happens, one could expect to discover some adverse effect signal that is in the background of the increasing trend of adverse events reporting.

In the study of adverse events reporting rate trends (2004–2021) associated with the use of selective serotonin reuptake inhibitors (SSRI) significantly increasing trends were observed for arrhythmias and QT interval prolongation. Further disproportionality analysis of the same database confirmed the existence of signals for arrhythmias and QT interval prolongation for all investigated SSRIs [14]. This example underlines the importance of analyzing adverse drug events reporting trends as a pilot for the initiation of investigating signals. We should also bear in mind that serious adverse drug reactions become more readily spotted only several years after marketing authorization approval, due to their inherent rarity and idiosyncrasy [25]. Whether the increasing trend of semaglutide serious adverse events reporting observed in our study is linked to some safety signal(s) remains to be explored in future studies.

There are several limitations of our study. First, only one adverse event reporting database was used for analysis, which may introduce bias due to specific construction characteristics of that database, different from others. Second, not all anti‐obesity drugs could have been followed for a sufficient number of years (due to their recent marketing authorization) decreasing the overall statistical power of the trend analysis. Third, the database analyzed did not have the option to construct separate trends according to the approved indications for which the drugs were used, which caused clumping of the adverse event reports and possible bias due to possible off‐label use of some of the drugs.

In conclusion, among the anti‐obesity drugs currently approved, only semaglutide shows a continuously increasing trend of annual reporting of serious adverse events, suggesting a need for further investigation of safety signals.

## Author Contributions

Project administration: Andrej Belančić, Slobodan M. Janković. Methodology: Slobodan M. Janković, Branislava B. Raičević. Investigation: Slobodan M. Janković, Andrej Belančić, Branislava B. Raičević, Nikola Mirković. Writing – original draft: Branislava B. Raičević, Slobodan M. Janković. Writing – review and editing: Branislava B. Raičević, Andrej Belančić, Nikola Mirković, Slobodan M. Janković. Conceptualization: Slobodan M. Janković. Supervision: Slobodan M. Janković. All authors have read and agreed to the published version of the manuscript.

## Ethics Statement

The authors have nothing to report.

## Conflicts of Interest

The authors declare no conflicts of interest.

## Data Availability

Available upon reasonable request sent to the corresponding author.
